# The Cause and Prevention of Dental Decay

**Published:** 1900-02

**Authors:** Geo. Howe Winkler

**Affiliations:** New York.


ARTICLE IV.

### THE CAUSE AND PREVENTION OF DENTAL DECAY.

GEO. HOWE WINKLER, M. D., D. D. S., NEW YORK.

Read before the New Jersey State Dental Society.

The cause of dental decay is a subject that has been so much speculated upon and theorized about that the discussion of it seems almost like the threshing over of old straw; yet the importance of the subject, especially in the light of recent publications, justifies us in continuing our investigations, more especially as we know that in the searching over of old materials new values are sometimes discovered. The farmers of the south for over one hundred years used the cottonseed for fertilizing purposes only, not realizing that the oil which these seeds contained constituted a crop worth several millions of dollars annually.

The various theories that have been held in regard to dental decay, involving the ideas of inflammation in the organic matrix of the tooth, as well as the bacterial destruction, have been advanced and maintained by their several advocates in arguments so convincing as to insure for each theory a large following. But these theories are incorrect, because they are conclusions drawn from the evidences found in the mouth where dental decay is in a state of progress and not in its incipiency.

The microscopic and clinical researches of Prof. Miller, of Berlin, and of Dr. Leon Williams, of London, and the profound and extensive experiments and deductions of Dr. G. V. Black, of Illinois, have fixed in the minds of most members of the profession the idea that acid producing bacteria are the cause of decay. There is no doubt that the very able experments and writings of Prof. Miller were absolutely accurate in stating conditions as he found them in the mouth, and the marvelously beautiful microscopic slides of Dr. Leon Williams show bacteria in their destructive work, but the conclusions which these gentlemen drew I am sure are wrong.

We know from their teachings that bacteria excreting acid and boring into the tubuli of the teeth are to be found in nearly all cavities of decay, but they are also to be found sometimes in limited numbers, and oftentimes of inoffensive character, in nearly all human mouths. Dr. Black engaged in the most extensive series of experiments and carried them out with a masterful intensity of purpose, and from the results drew conclusions that constitute, in my opinion, a most profound and able contribution to the literature of dentistry. Starting his research from the conditions in the mouth which are within the reach of human knowledge—chemical, microscopic—tangible—he worked up to the very portals of truth, when in speaking of the apparent lack of defensive proteids in the mucous secretions and saliva of some persons, and of the lack of the proteids at some periods and not at others in the same person, and especially of some ages, as of the years of puberty and of periods of pregnancy, he says: "Taking this view of the condition" (that is that the lack or presence of defensive proteids in the secretions of the mouth account for the destruction of the healthy integrity of the teeth, and that lack or presence is due to a constitutional condition) "dental caries becomes as truly a dyscrasia as rheumatism or gout," and he was absolutely right.

I purpose to show why I believe he was right, and to place it in the power of every dentist present to prove the truth of his conclusion in that particular.

### DENTAL CARIES DUE TO DYSCRASIA.

Dental decay is due to a dyscrasia, which can be recognized in its incipiency by some of its symptoms only. The evidence of decay in the tooth is not the cause of decay, it is but the progress of the disease—the recognizable stage of the morbid condition, just as the eruption on a fever patient defines the character of the patient's sickness. The cause of dental decay is a condition in the system that is too subtle for human knowledge to grasp. The earliest symptoms of the dyscrasia that we can detect are discovered in the perverted secretions of the oral cavity, by means of litmus paper when acidity prevails or by the physical appearance of the fluids there, such as slimy deposits on the teeth, or saliva which is thick, ropy, gluey, as well as acid—sometimes by swollen, spongy, bleeding gums accompanying white decay—a disagreeable taste in the mouth in the morning, and at times an offensive odor on the breath. Similar stages of disease exist in the case of many sicknesses. A person sick with small-pox has the disease recognized when the eruption has become well developed, but the cause of that eruption was certainly in the system when the patient first began to feel droopy, or exhibited a slight condition of fever. The skin destroying pustules on the surface were not the cause of the smallpox. They simply represent a stage in the progress of the disease, which defines or classifies the dyscrasia. So it is with decay of the teeth. When we are able to see it, and under microscope find the acid-producing bacteria performing their functions—that is a stage in the disease, but not the cause of it.

### TREATMENT FOR PREVENTION OF DENTAL CARIES.

The treatment of the dyscrasia which is the cause of dental decay is marked in some instances with such remarkable curative results and benefit that it simply corroborates what is here laid down, and elevates my deduction and statements from the realm of theory to the plane of fact. I am advancing no theory. I am simply stating facts which can be corroborated and proven by any one who will follow the accompanying directions for treatment. These conditions in the mouth must be treated from within, because they are not due to a diseased condition of the mucous glands or the salivary glands, but to the condition of the blood or the abnormal state in the system. The only way that the mucous glands could be arrested in their excretion of these viscid and foul exudations would be by destroying them, and that would not relieve the system of the condition. These glands in throwing out the perverted and viscous secretions are only performing the natural function of elimination, and they will continue to perform that function as long as the blood contains these poisonous products, and they themselves retain their health and power to work. When chlorate of potash, for instance, is administered internally, in a few minutes after the administration the drug can be found by tests in the saliva. That is certainly not due to any defect, or any disease in the glands, but to the condition in the blood, and the evidence of it in the saliva only ceases when the potash has been entirely eliminated. The treatment for the prevention of decay must be a treatment directed to the cure of the systematic condition, which is the incipient stage of the disease, and we must be guided in that treatment by the symptoms presented in the mouth. I administer internally for the prevention of decay but four remedies. These I give in homœopathic triturations, and generally in what is known as potencies—the two-hundredth being my favorite.

For corrosive acid excretions from the mucous glands which disintegrate and destroy teeth with a rapidity greater than can be produced by the aid of micro-organisms, the homœopathic trituration of creosote is a specific, administered in small doses, eight or ten doses per day at intervals of an hour when a low potency, such as creosotum 6, is used. If given in very high potency the remedy can be administered in one dose at night upon retiring, and one dose in the morning upon rising and before eating. The creosotum, in addition to immediately arresting the acid excretions of the mouth, produces a most wonderful and beneficent action upon the organic matrix of the tooth. Given night and morning in high potency to patients whose teeth are exquisitely sensitive it greatly relieves the sensitiveness, so that dental operations are rendered almost painless within a week's time while the dentine seems harder under the instrument.

For the white or light brown decay, found in the mouths of children or patients in their teens, and of pregnant women, the trituration of lime, carbonate of lime, calcarea carb., or phosphate of lime, calcarea phos., in alternation with creosote is wonderfully efficacious.

The carbonate of lime is the more valuable of the two lime preparations, and is designed more especially for patients who are inclined to be fair and plump with light eyes and hair. The phosphate of lime being more especially indicated in patients who are dark as to their complexion, hair and eyes, and tense in their fiber, rather inclined to be thin.

Carbo veg., or vegetable charcoal, in homœopathic trituration, is a magnificent remedy for patients suffering from swollen, spongy gums, which bleed at the slightest touch.

The appearance of the mouth in this class of etiological factors shows generally very slight inflammation, although the gums are sometimes swollen and bleed very readily, the entire mouth being bathed with a mild acid except after some minutes of mastication whereby the alkaline saliva has overcome the acid, and the teeth are affected by decay, more or less extensive, which is characterized by a very light brown or chalky white color, and by rapid progress, the patient generally being anæmic. The charcoal trituration not only relieves the mouth of its conditions, but brings a healthy color to cheeks and lips, proving itself a wonderful tonic to the blood.

The bichromate of potash or kali bich., is a specific for ropy, gluey, stringy, acid saliva, which pulls out in strings on one's fingers, and can only be expectorated with an effort.

I prefer as a rule to use the highest potencies I can secure, because their action is much more speedy than the lower potencies, and their administration is much more convenient.

A few granules of the specified remedy poured from a vial into the hand of the patient, and then taken on the tongue night and morning, is sufficient if the potency or trituration is as high as the two-hundredth or higher, and more frequently if the triturations are in the decimals, like 6x or 12x.

In conclusion I will recapitulate my statements :

The cause of dental decay is a dyscrasia or morbid state in the system.

It can be recognized in its early stages or incipiency by the perverted secretions in the mouth.

At this stage the dyscrasia is capable of successful treatment and cure by medicines internally administered.

The remedies neccessary to cure the dyscrasia and thus prevent dental decay are but four in number, and must be in the form of homœopathic triturations.

They are creosote, creosotum, for corrosive acid excretions.

Lime carbonate or phosphate—calcarea carb., or calcarea phos., for the light brown or white decay of childhood, puberty and pregnancy.

The bichromate of potash—kali bich., for ropy, gluey, viscid and acid saliva.

And vegetable charcoal—carbo. veg., for white decay, mild acidity of saliva, and swollen, spongy, easily bleeding gums, the patient presenting anæmic appearance.

This is my message to you to-day. It is not a theory. It a simple statement of facts; the truth of which can be easily demonstrated by every practitioner who will apply intelligently the above simple remedies. My own experience convinces me that at least fifty per cent. of dental decay can be prevented by the internal administration of these medicines.—*Items of Interest.*

